# Chondrogenic induction of mesenchymal stromal/stem cells from Wharton’s jelly embedded in alginate hydrogel and without added growth factor: an alternative stem cell source for cartilage tissue engineering

**DOI:** 10.1186/s13287-015-0263-2

**Published:** 2015-12-30

**Authors:** Loïc Reppel, Jessica Schiavi, Naceur Charif, Léonore Leger, Hao Yu, Astrid Pinzano, Christel Henrionnet, Jean-François Stoltz, Danièle Bensoussan, Céline Huselstein

**Affiliations:** UMR 7365 CNRS-Université de Lorraine, Ingénierie Moléculaire et Physiopathologie Articulaire (IMoPA), Biopôle, 54505 Vandœuvre-lès-Nancy, France; CHU de Nancy, Unité de Thérapie Cellulaire et Tissulaire, 54500 Vandœuvre-lès-Nancy, France; Université de Lorraine, 54000 Nancy, France; Fédération de Recherche 3209, Bioingénierie Moléculaire Cellulaire et Thérapeutique, 54500 Vandœuvre-lès-Nancy, France

**Keywords:** Alginate/hyaluronic acid hydrogel, Chondrogenic differentiation, Cartilage tissue engineering, Mesenchymal stromal/stem cells, Wharton’s jelly

## Abstract

**Background:**

Due to their intrinsic properties, stem cells are promising tools for new developments in tissue engineering and particularly for cartilage tissue regeneration. Although mesenchymal stromal/stem cells from bone marrow (BM-MSC) have long been the most used stem cell source in cartilage tissue engineering, they have certain limits. Thanks to their properties such as low immunogenicity and particularly chondrogenic differentiation potential, mesenchymal stromal/stem cells from Wharton’s jelly (WJ-MSC) promise to be an interesting source of MSC for cartilage tissue engineering.

**Methods:**

In this study, we propose to evaluate chondrogenic potential of WJ-MSC embedded in alginate/hyaluronic acid hydrogel over 28 days. Hydrogels were constructed by the original spraying method. Our main objective was to evaluate chondrogenic differentiation of WJ-MSC on three-dimensional scaffolds, without adding growth factors, at transcript and protein levels. We compared the results to those obtained from standard BM-MSC.

**Results:**

After 3 days of culture, WJ-MSC seemed to be adapted to their new three-dimensional environment without any detectable damage. From day 14 and up to 28 days, the proportion of WJ-MSC CD73^+^, CD90^+^, CD105^+^ and CD166^+^ decreased significantly compared to monolayer marker expression. Moreover, WJ-MSC and BM-MSC showed different phenotype profiles. After 28 days of scaffold culture, our results showed strong upregulation of cartilage-specific transcript expression. WJ-MSC exhibited greater type II collagen synthesis than BM-MSC at both transcript and protein levels. Furthermore, our work highlighted a relevant result showing that WJ-MSC expressed Runx2 and type X collagen at lower levels than BM-MSC.

**Conclusions:**

Once seeded in the hydrogel scaffold, WJ-MSC and BM-MSC have different profiles of chondrogenic differentiation at both the phenotypic level and matrix synthesis. After 4 weeks, WJ-MSC, embedded in a three-dimensional environment, were able to adapt to their environment and express specific cartilage-related genes and matrix proteins. Today, WJ-MSC represent a real alternative source of stem cells for cartilage tissue engineering.

## Background

Once damaged, cartilage tissue has limited self-repair capacity. Today, traumatic and degenerative articular cartilage damage can only be treated symptomatically (analgesics and anti-inflammatory drugs) or by surgery (mosaicoplasty, microfracture, autologous chondrocyte implantation) in order to delay joint replacement. However, these methods fail to restore native tissue integrity and lead to the formation of fibrocartilage [1] which is functionally inferior to hyaline cartilage. For these reasons, scientists and clinicians consider cartilage tissue engineering to be a potential alternative treatment for cartilage repair. Tissue engineering uses three basic elements: a suitable cell source, a biocompatible scaffold and environmental factors [2] to produce in vitro or in situ neotissue. These three elements can be combined or used separately to repair cartilage defect. Several investigators preferred transplantation of cells only combined with scaffold to create functional tissue replacement in situ [3]. Three-dimensional (3D) scaffolds must be able to mimic the physiological environment and ensure attachment, proliferation and differentiation of cells. Due to their intrinsic properties, stem cells are promising tools for new tissue engineering developments and particularly for cartilage tissue regeneration. Owing to ethical considerations and the random efficiency of chondrogenic differentiation [4], the use of embryonic stem cells is not the most appropriate. Thus, mesenchymal stromal/stem cells (MSC) are an attractive source of cells for cartilage tissue engineering.

MSC from bone marrow (BM-MSC) remain the most studied stem cell source used in cartilage tissue engineering [5, 6]. However, bone marrow collection is a painful and invasive procedure with the possibility of donor site damage. In addition, it has been demonstrated that the number of available BM-MSC is quite low in this compartment [7], and their differentiation potential and proliferation capacity decrease with age [8, 9]. Consequently, the use of autologous BM-MSC for tissue repair, which in some indications concerns elderly patients, has certain limits. Thus, identifying alternative sources of MSC would be very helpful.

Due to their properties such as low immunogenicity [10] and, particularly, chondrogenic differentiation potential [11], MSC from the connective tissue of umbilical cord named Wharton’s jelly (WJ-MSC) promise to be an interesting source of MSC for cartilage tissue engineering [12]. Several studies have already demonstrated the potential of WJ-MSC for chondrogenic differentiation in 3D cultures. WJ-MSC were embedded in natural scaffolds such as type I collagen hydrogel [13] or in synthetic polymer scaffolds such as polyglycolic acid meshes [14], and polyvinyl alcohol-polycaprolactone [15]. Cells were cultivated for 3 to 4 weeks in chondrogenic medium supplemented with growth factors (such as transforming growth factor (TGF)-β1 and TGF-β3 and bone morphogenic protein (BMP)2) used alone or in combination [15]. These works showed the successful chondrogenic induction of WJ-MSC in 3D scaffolds with expression of specific cartilage-related genes and matrix proteins (Sox9, aggrecan, type II collagen, and cartilage oligomeric matrix protein (COMP)).

Alginate hydrogel is an in vitro and in vivo biocompatible scaffold [16], and a hydrophilic polymer network which creates a porous microstructure ensuring nutrient diffusion, cell to cell contact, cell proliferation and differentiation [17]. Various studies have already shown that MSC embedded in alginate hydrogel represent a relevant model for chondrogenesis of human MSC and study of the molecular mechanisms involved in chondrogenic differentiation [6, 17]. Hyluronic acid (HA) is a natural component of native cartilage and HA hydrogel can support and promote the chondrogenic differentiation of MSC [18]. According to a recent in vivo study, HA is an attractive hydrogel candidate for cartilage tissue engineering [19].

In this study, we propose to evaluate chondrogenic potential of WJ-MSC embedded in alginate/hyaluronic acid (Alg/HA) hydrogel. Hydrogels were constructed by an original spraying method which has been previously described [6, 20]. Our main objective is to evaluate chondrogenic differentiation of WJ-MSC in a 3D scaffold, without adding growth factors, at transcript and protein levels. To conclude whether WJ-MSC represent a real alternative source of stem cells for cartilage tissue engineering, we compared the results to those obtained from standard BM-MSC.

## Methods

### Isolation and culture of MSC

WJ-MSC and BM-MSC were isolated and cultivated as previously described [12]. Human umbilical cords and bone marrow were collected after patients’ informed consent; this complied with national legislation regarding human sample collection, manipulation and personal data protection. These biological samples were regarded as surgical waste and therefore, following the opinion of an ethics committee of Nancy Hospital, no authorization of this committee was necessary for their collection. BM-MSC were only used as a standard control.

Umbilical cord samples, from three donors, were rinsed with 70 % ethanol and Hanks’ balanced salt solution (HBSS). To perform MSC isolation, the umbilical cord vessels were removed and Wharton’s jelly aseptically cut into small pieces (2 to 3 mm^3^) which were plated in a six-well plate with complete medium (minimal Eagle medium (α-MEM; Lonza, Walkersville, MD, USA) with 10 % fetal bovine serum, glutamine 2 mM, penicillin 100 IU/mL, streptomycin 100 μg/mL and amphotericin B 2.5 μg/mL). They were incubated at 37 °C under a humidified atmosphere with 5 % CO_2_ in normoxia. After 7 days of contact with the plastic surface the cells migrated and, as enough adherent cells were obtained, the pieces were removed, the medium replaced and cultures continued until cell subconfluence (80–90 %). After 2 weeks, WJ-MSC were harvested with 0.25 % Trypsin-EDTA (Sigma-Aldrich, St. Louis, MO, USA) and grown up to passage (P)3.

Bone marrow samples from five donors aged from 25 to 60 years were aspirated and diluted in HBSS. Nuclear cells were counted and the cell suspension was seeded at 50,000 nuclear cells/cm^2^ with complete medium. They were incubated at 37 °C under a humidified atmosphere with 5 % CO_2_ in normoxia. BM-MSC migrated, adhered to the plastic surface and were cultivated up to subconfluence (P0). After 2 to 3 weeks, cells were harvested with Trypsin-EDTA, seeded with complete medium at 1000 cells/cm^2^ and grown up to P3.

### Scaffold construct and chondrogenic differentiation

**Fig. 1:**
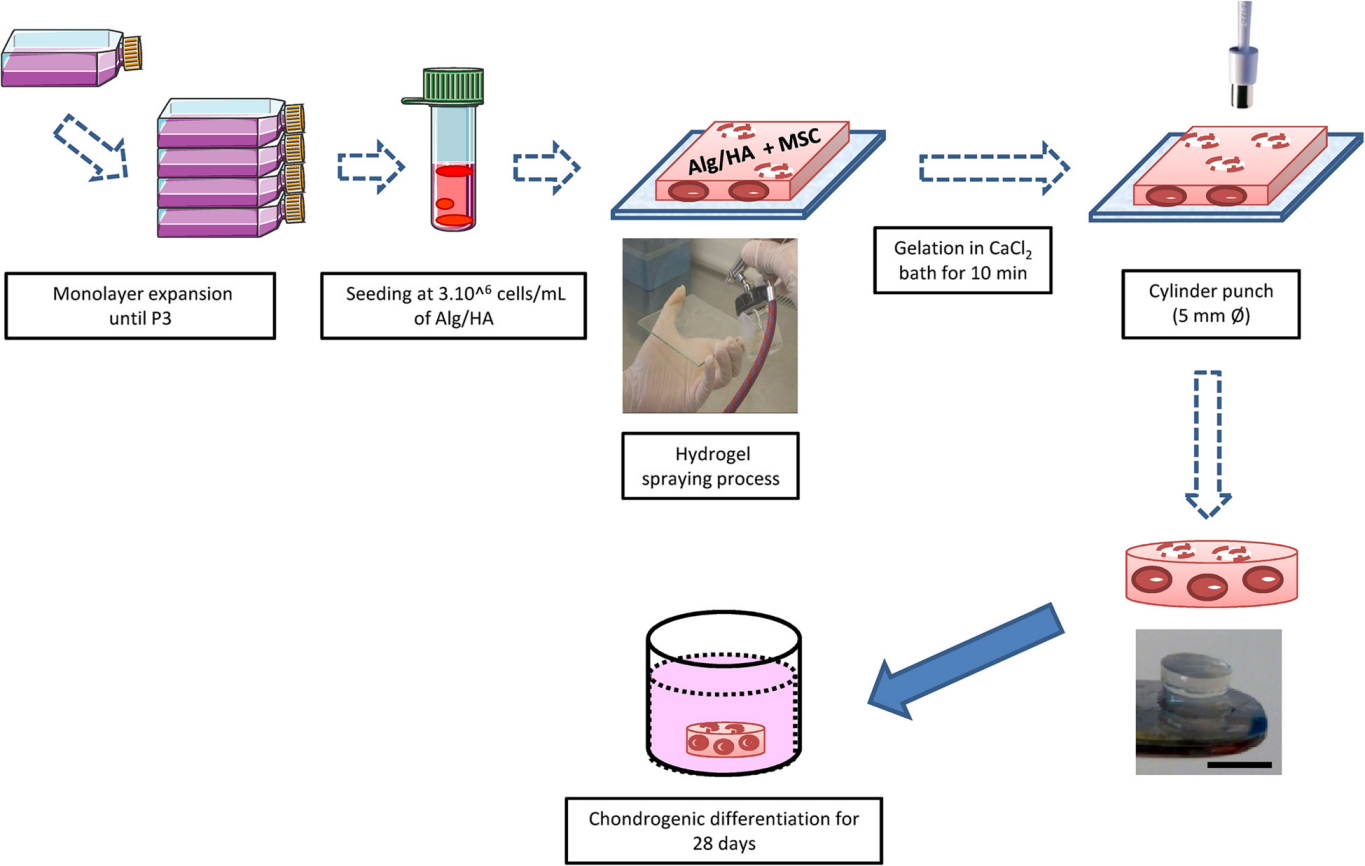
Illustration of protocol steps used to perform scaffold construct and chondrogenic differentiation. After monolayer expansion, MSC were seeded at 3× 10^6^ cells/mL of Alg/HA hydrogel. Hydrogel was sprayed, gelated, and cut into 5 mm diameter cylinders; scale bar = 5 mm. Scaffolds were cultivated in a 48-well plate in differentiation medium for 28 days. *Alg/HA* alginate/hyaluronic acid, *MSC* mesenchymal stromal/stem cells, *P3* passage 3

After expansion, at the end of the third passage, WJ-MSC and BM-MSC were harvested with Trypsin-EDTA and seeded at 3 × 10^6^ cells/mL of Alg/HA hydrogel (Fig. 1). Scaffolds were built up with one hydrogel layer seeded with MSC. Hydrogel was composed of 1.5 % (m/v) Alg (medium viscosity, Sigma, France) and HA (Accros, France) (ratio 4:1) dissolved in 0.9 % NaCl. The spraying method used was previously described with rat chondrocytes [20] and human MSC [6]. The spraying system consisted of an airbrush working with a compressor to induce spraying, with pressure being equal to 0.9 bar. The spraying bottle containing the homogenized cellular suspension in Alg/HA hydrogel was connected to the airbrush, and then solution was sprayed on a sterile glass plate. After hydrogel gelation in a CaCl_2_ bath at 102 mM for 10 minutes, cylinders were cut at 5 mm diameter and 2 mm thickness with a biopsy punch (Fig. 1).

For viability analysis, WJ-MSC were also seeded in Alg/HA hydrogel beads, manufactured as previously described [21], by simple encapsulation without spraying and used as a control.

Scaffolds were cultivated in a 48-well plate in differentiation medium containing DMEM-high glucose (Gibco, Grand Island, NY) supplemented with 10 % fetal bovine serum (Gibco), glutamine 2 mM (Sigma), penicillin 100 U/mL (Sigma), streptomycin 100 μg/mL (Sigma), amphotericin B 2.5 μg/mL (Sigma), 100 μg/mL sodium pyruvate (Sigma), 40 μg/mL L-Proline (Sigma), 50 μg/mL L-acid ascorbic (Sigma), 100 nM dexamethasone (Sigma) and 1 mM CaCl_2_ (Sigma). Scaffolds were incubated at 37 °C under a humidified atmosphere with 5 % CO_2_ in normoxia for 28 days and differentiation medium was changed twice a week (Fig. 1).

During chondrogenic differentiation, after 3, 14 and 28 days of culture, cells were extracted from Alg/HA hydrogel by dissolution in 55 mM sodium citrate (Sigma) and 50 mM EDTA solution (Merck, Darmstadt, Germany) for 5 minutes. After centrifugation (320 g, 5 minutes), viability, phenotype and mRNA expression were analyzed. Only at 28 days of culture were other scaffolds used for histological processing. WJ-MSC viability was also evaluated for two methods of scaffold construct: alginate beads and alginate cylinders (obtained by spraying method) at 3, 7 and 10 days of culture.

### Viability, apoptosis and necrosis analysis

**Fig. 2:**
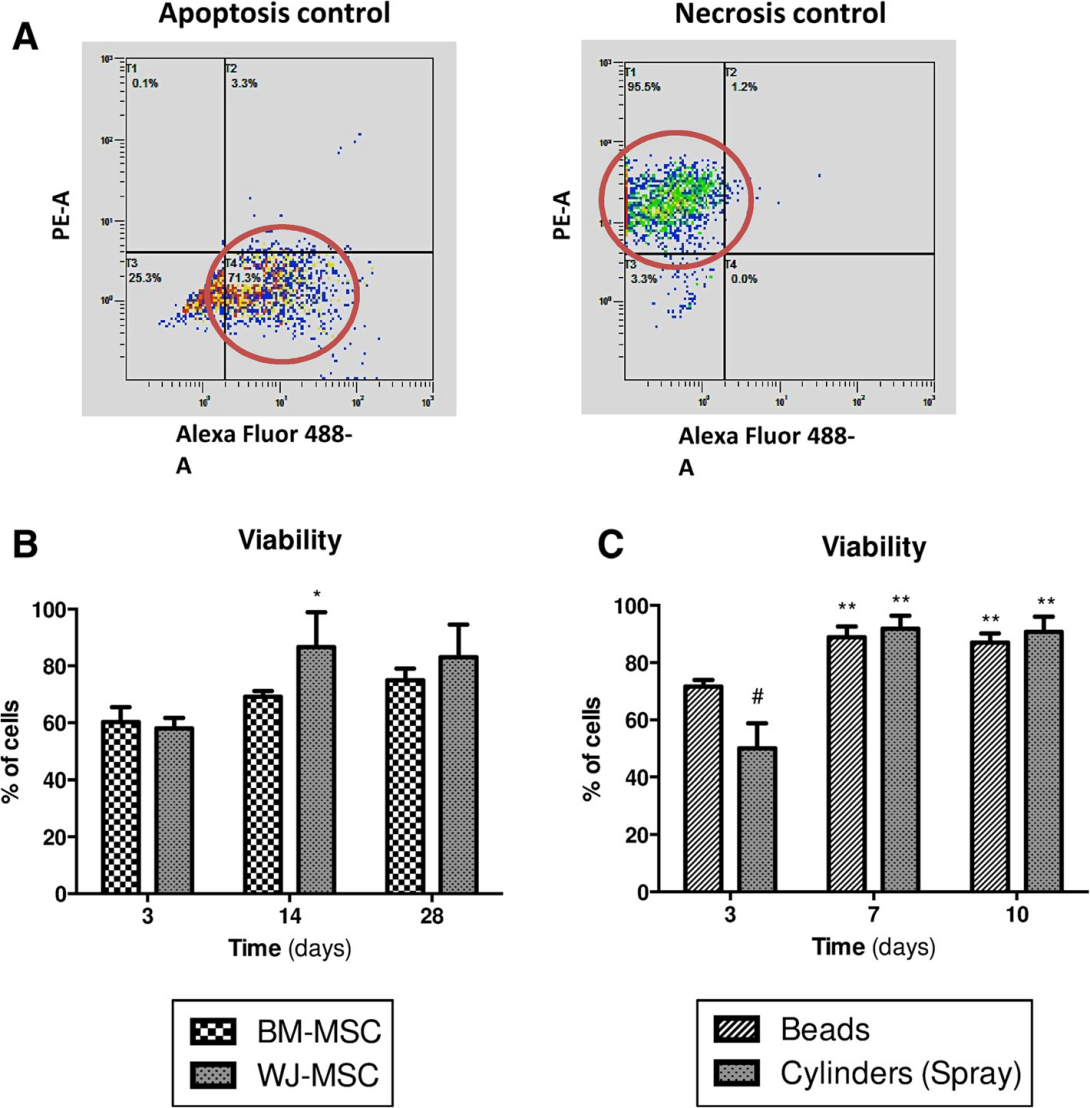
Changes in MSC viability during scaffold culture. Cell viability was measured by flow cytometry at 3, 14 and 28 days of culture of MSC embedded in Alg/HA hydrogel. Necrotic and apoptotic cells were labeled with propidium iodide and annexin V–Alexa 488, respectively. **a** Positive controls for apoptotic and necrotic cells. **b** Cell viability was evaluated after spraying method of scaffold construct between BM-MSC and WJ-MSC. **c** WJ-MSC viability was evaluated for two methods of scaffold construct: alginate beads and alginate cylinders (obtained by spraying method) at 3, 7 and 10 days of culture. The results are expressed as mean ± standard error of the mean (*n* ≥ 3). *p < 0.05 and **p < 0.01, day x vs day 3 for the same cell source (**b**) or method of scaffold construct (**c**). ^#^ *p* < 0.05, cylinders vs beads for the same culture time. *BM-MSC* bone marrow-derived mesenchymal stromal/stem cells, *WJ-MSC* Wharton’s ielly-derived mesenchymal stromal/stem cells

Apoptosis and necrosis of cells were analyzed by flow cytometry using the Vybrant/Apoptosis^TM^ kit based on the AnnexinV/propidium iodide (PI) staining procedure (Invitrogen, Carlsbad, CA). Cells were suspended in 100 μL 1× Annexin-liant buffer with 2.5 μL Annexin V-Alexa 488 and 1 μL PI (100 μg/mL), for 15 minutes at room temperature. After incubation, 200 μL 1× Annexin V buffer were added to each sample. Then, cells were analyzed by measuring fluorescence emission at 530 nm and 575 nm, respectively, for Alexa 488 (apoptotic cells) and PI (necrotic cells) with a Gallios flow cytometer (Beckman Coulter, Brea, CA, USA). Negative (unlabeled cells) and positive controls (apoptosis and necrosis) were performed (Fig. 2a). Apoptosis was induced by incubating anti-Fas mouse antibody (Enzo Life Sciences, Farmingdale, NY, USA) with the cells for 1 hour. After washing, a second anti-mouse IgG3 antibody (BD, Franklin Lakes, NJ, USA) was incubated for 1 hour with cells and, finally, cells were labeled only with Annexin V-Alexa 488. Cell necrosis was induced by adding Triton X-100 solution (Sigma) for 1 minute before centrifugation (300 g, 5 minutes) and PI labeling. For all analyses, at least 5000 events were analyzed. Viable cells were Annexin V ^−^ and PI ^−^.

### Phenotypic analysis

Phenotypic analysis of MSC was performed during monolayer expansion (prior to encapsulation in the hydrogel) and throughout scaffold culture. Briefly, to perform phenotypic analysis MSC were incubated with fluorescein isothiocyanate (FITC)- or phycoerythrin (PE)-conjugated mouse anti-human antibodies CD34-PE, CD45-FITC, HLA-DR-FITC, CD44-FITC, CD73-PE, CD90-FITC, CD105-PE and CD166-PE (Beckman Coulter, Brea, CA, USA) for 30 minutes at room temperature. Negative and isotype (FITC and PE) controls were performed. After immunofluorescence staining, for each sample 10,000 events were counted by Gallios flow cytometer (Beckman Coulter, Brea, CA, USA).

### Transcript analysis

MSC were rinsed with phosphate-buffered saline three times to remove residual alginate. Total RNA was extracted by RNeasy Plus mini kit (Qiagen, Hilden, Germany) according to the manufacturer’s instructions. RNA yield was evaluated by spectrophotometry and RNA quality was analyzed by electrophoresis through a 1 % agarose gel. RNA was then reverse-transcripted to cDNA using iScript™ cDNA Synthesis Kit (Bio-rad, Hercules, CA, USA). Quantitative polymerase chain reaction (PCR) was performed using iTaq™ Universal SYBR® Green Supermix (Bio-rad) and a Light Cycler system (Roche Diagnostics, Basel, Switzerland) during 45 cycles to quantitatively analyze gene expression. Values were normalized to expression of RP29 mRNA. Table 1 lists the specific primers used.

**Table 1:** List of polymerase chain reaction primers used for the present study

Name	Forward primer (5′–3′)	Reverse primer (5′–3′)	TM (°)
RP29	AAGATGGGTCACCAGCAGCTCTACTG	AGACGCGGCAAGAGCGAGAA	60
Sox9	GAGCAGACGCACATCTC	CCTGGGATTGCCCCGA	55
Aggrecan	TCGAGGACAGCGAGGCC	TCGAGGGTGTAGCGTGTAGAGA	62
COMP	ACAATGACGGAGTCCCTGAC	TCTGCATCAAAGTCGTCCTG	60
Type IIa collagen	GCAGGATGGGCAGAGGTAT	ATCTCAGGGCTGAGGCAGT	60
Total II collagen	ATGACAATCTGGCTCCCAAC	GAACCTGCTATTGCCCTCTG	55
Type X collagen	GCTAAGGGTGAAAGGGGTTC	CTCCAGGATCACCTTTTGGA	60
Runx2	CCCGTGGCCTTCAAGGT	CGTTACCCGCCATGACAGTA	56
PPAR γ	TTCAGAAATGCCTTGCAGTG	CCAACAGCTTCTCCTTCTCG	58

### Matrix synthesis

Matrix synthesis was evaluated by histology, immunofluorescence staining and immunohistochemistry. After 28 days of chondrogenic differentiation, Alg/HA scaffolds were fixed in 4 % paraformaldehyde (Sigma), 100 mM sodium cacodylate (Sigma) and 10 mM CaCl_2_ (Sigma) solution (pH 7.4) for 4 hours and then washed overnight in 100 mM sodium cacodylate and 50 mM BaCl_2_ (Sigma) buffer (pH 7.4). The scaffolds were dehydrated, embedded in paraffin blocks, cut into 5 μm thick sections and mounted onto glass slides.

For histological analysis, total collagen and proteoglycans were stained by Sirius red and Alcian Blue, respectively, and observed by light microscopy (DMD 108, Leica, Wetzlar, Germany).

For immunofluorescence staining, hydrogel sections were deparaffinized and permeabilized with 0.1 % Triton X-100 (Sigma) for 20 minutes and then blocked with 0.5 % bovine serum albumin (Sigma) in DMEM without phenol red (Gibco) for 15 minutes at room temperature. Specimens were incubated for 45 minutes with rabbit anti-human type I, II, or X collagen antibodies (1:50) (Merck, Darmstadt, Germany). After a washing step, samples were incubated for 45 minutes with secondary antibody: a goat anti-rabbit IgG Alexa Fluor 488 (1:50) (Invitrogen, Carlsbad, CA, USA). Negative and isotype controls were also performed. Immunofluorescence labeling was detected using fluorescence microscopy (DMI 3000B, Leica, Wetzlar, Germany).

Immunohistochemistry with antibodies for collagen type type I, II and X was performed according to LSAB® + kit (HRP, Dako) based on avidin-biotin techniques. Primary monoclonal antibodies collagen I (T59103R, Biodesign) and collagen II (6B3, Labvision) were used at the dilution of 1/100 and collagen X (Ab49945, Abcam) was used at the dilution of 1/1000. Paraffin-embedded tissue sections of 5 μm were deparaffinized through a series of alcohols and treated with pepsin (0.4 % w/v in 0.01 M HCl, pH 2.0, Sigma) for 30 minutes at room temperature. Slides were then incubated with hydrogen peroxide block solution for 5 minutes to block endogenous peroxidase. After washing, 2 % bovine serum albumin solution was applied for 10 minutes at room temperature to block the unspecific epitope. The primary antibody was added to each slide and slides were incubated at room temperature in a humidified chamber for 1 hour. Subsequently, the samples were incubated with a biotinylated link secondary antibody for 45 minutes at room temperature. Peroxidase-labeled steptavidin was applied at room temperature for 30 minutes. Substrate-chromogen solution was prepared with diaminobenzidine (DAB, LSAB® + kit, Dako) and incubated to the specimen and monitored under a microscope for the desired stain intensity. Control groups for immunohistochemical analysis were performed under identical conditions on human cartilage for positive control or without primary antibodies for negative control. Finally the sections were counterstained with hematoxylin at 1/5 for 1 minute (RAL, France) and mounted with Eukit resin.

### Statistical analysis

Statistical tests and graphic representations were performed using graphPad Prism 5 software (GraphPad, San Diego, CA, USA). All the data are presented as mean ± standard error of the mean of independent experiments with cells from different donors that were pooled. Significant statistical differences were calculated using one- or two-way analysis of variance. A *p*-value less than 0.05 was considered significant for the analyses of variance. If significance existed, a post-hoc analysis was performed using the Bonferonni post-tests to evaluate significance for all experiments.

## Results

### Viability, apoptosis and necrosis analysis

Cell viability, apoptosis and necrosis were analyzed 3, 14 and 28 days after spraying method of scaffold construct and data were compared to BM-MSC (Fig. 2b). After 3 days of culture, WJ-MSC viability was 58 ± 4 %. From 14 days, viability significantly increased (*p* < 0.05) and became greater than 80 % until 28 days. Meanwhile, apoptosis and necrosis decreased from 14 up to 28 days (data not shown). No significant difference was observed between WJ-MSC and BM-MSC. To understand WJ-MSC behavior in early culture, the same parameters were evaluated using two methods of scaffold construct, alginate beads and alginate cylinders obtained by spraying method, between 3 and 10 days after build-up (Fig. 2c). The results indicated clearly that at the third day of culture the viability of sprayed cells was significantly lower than in cells embedded in alginate beads (*p* < 0.05). At the same time, cell apoptosis was significantly higher in cylinders compared to cells seeded in beads (*p* < 0.05) (data not shown).

### Phenotypic analysis

**Fig. 3:**
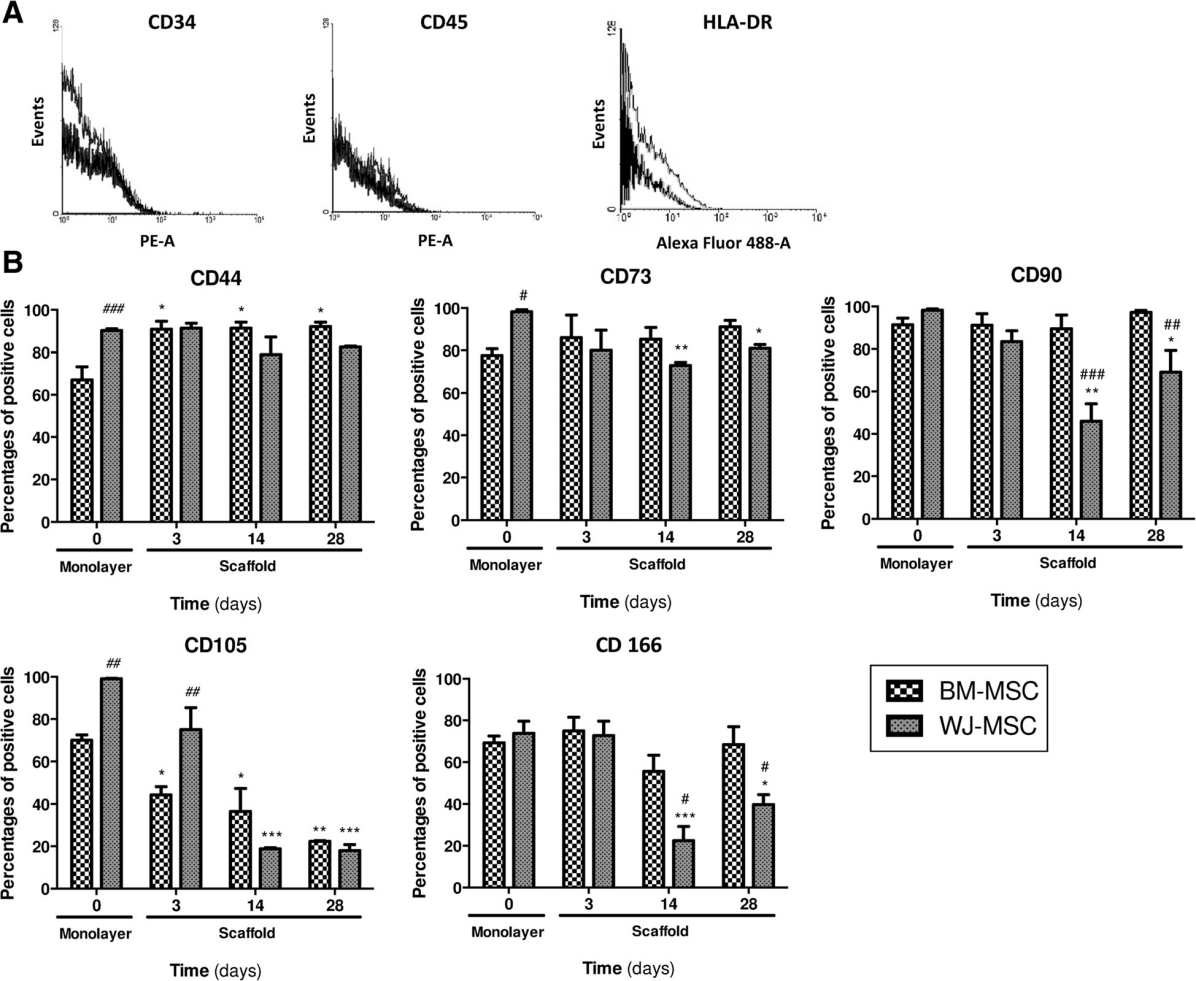
Immunophenotypic analysis of MSC by flow cytometry during monolayer expansion (prior to encapsulation in the hydrogel) and throughout scaffold culture. **a** Hematopoietic markers and major histocompatibility complex class II molecule. **b** Mesenchymal surface markers. For mesenchymal surface markers, the results are shown as percentages of positive cells. All results are expressed as mean ± standard error of the mean (*n* ≥ 3). *p < 0.05, **p < 0.01 and ***p < 0.001, day x vs day 0 (monolayer) for the same cell source. ^#^ *p* < 0.05, ^##^ *p* < 0.01 and ^###^ *p* < 0.001, WJ-MSC vs BM-MSC for the same culture time. *BM-MSC* bone marrow-derived mesenchymal stromal/stem cells, *WJ-MSC* Wharton’s jelly-derived mesenchymal stromal/stem cells

Flow cytometry showed that, regardless of the time of culture or the cell source, MSC were negative for the hematopoietic markers CD34, CD45 and HLA-DR (Fig. 3a). Less than 5 % of cells expressed these surface markers. WJ-MSC from the monolayer to the end of 3D culture variably expressed mesenchymal markers such as CD44, CD73, CD90, CD105 and CD166 (Fig. 3b). During monolayer expansion, the proportion of CD44^+^, CD73^+^ and CD105^+^ cells were significantly higher (at least 20 %) in WJ-MSC compared to BM-MSC (*p* < 0.001, *p* < 0.05 and *p* < 0.01, respectively). No difference was reported for CD90 and CD166 expression. In the 3D environment and during chondrogenic differentiation, the proportions of positive WJ-MSC for mesenchymal markers (except CD44) decreased significantly from 14 and up to 28 days, in comparison with monolayer expansion. On the other hand, the proportion of CD44^+^ cells remained relatively constant. From 14 days, positive expression for mesenchymal markers seemed to be less for WJ-MSC than for BM-MSC, especially for CD90 and CD166 which were significantly reduced (*p* < 0.05).

### Transcript analysis

**Fig. 4:**
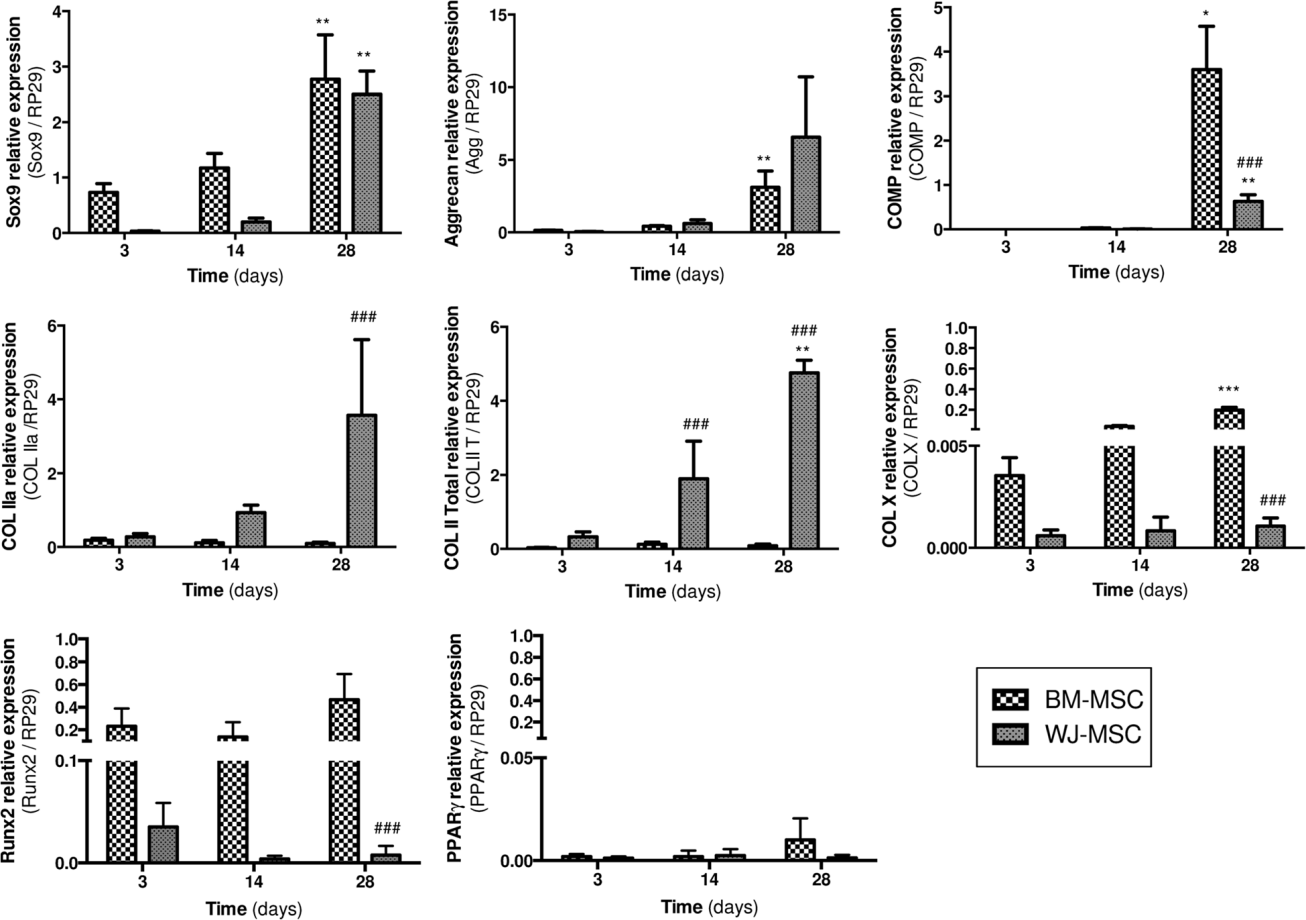
Relative expression of specific cartilage-related genes evaluated by quantitative RT-PCR during 28 days of chondrogenic induction. All results are expressed as mean ± standard error of the mean (*n* ≥ 3). *p < 0.05, **p < 0.01 and ***p < 0.001, day x vs day 3 for a same cell source. ^###^ *p* < 0.001, WJ-MSC vs BM-MSC for a same culture time. *BM-MSC* bone marrow-derived mesenchymal stromal/stem cells, *COMP* cartilage oligomeric matrix protein, *COL* collagen, *WJ-MSC* Wharton’s jelly-derived mesenchymal stromal/stem cells

Relative expression of specific cartilage-related genes was evaluated by quantitative RT-PCR during chondrogenic differentiation. Relative expression of other mesodermic lineage markers such as Runx2 or PPARγ was also reported (Fig. 4). While aggrecan and type IIa collagen expression seemed to increase during 3D culture of WJ-MSC, Sox9, COMP and total type II collagen expression by WJ-MSC were significantly higher compared to early stages of chondrogenesis (*p* < 0.01). After 28 days of chondrogenic induction, type IIa and total type II collagens were significantly more expressed by WJ-MSC than by BM-MSC (*p* < 0.001). Conversely, COMP expression was lower in WJ-MSC compared to standard BM-MSC (*p* < 0.001). Hypertrophic cartilage markers, Runx2 and type X collagen, were also analyzed during chondrogenic differentiation. Our results showed that Runx2 and type X collagen were very weakly expressed by WJ-MSC throughout culture compared to BM-MSC (*p* < 0.001 after 28 days of chondrogenic induction). Regardless of MSC source, no expression of adipogenic transcription factor PPARγ was detected during differentiation.

### Matrix synthesis

**Fig. 5:**
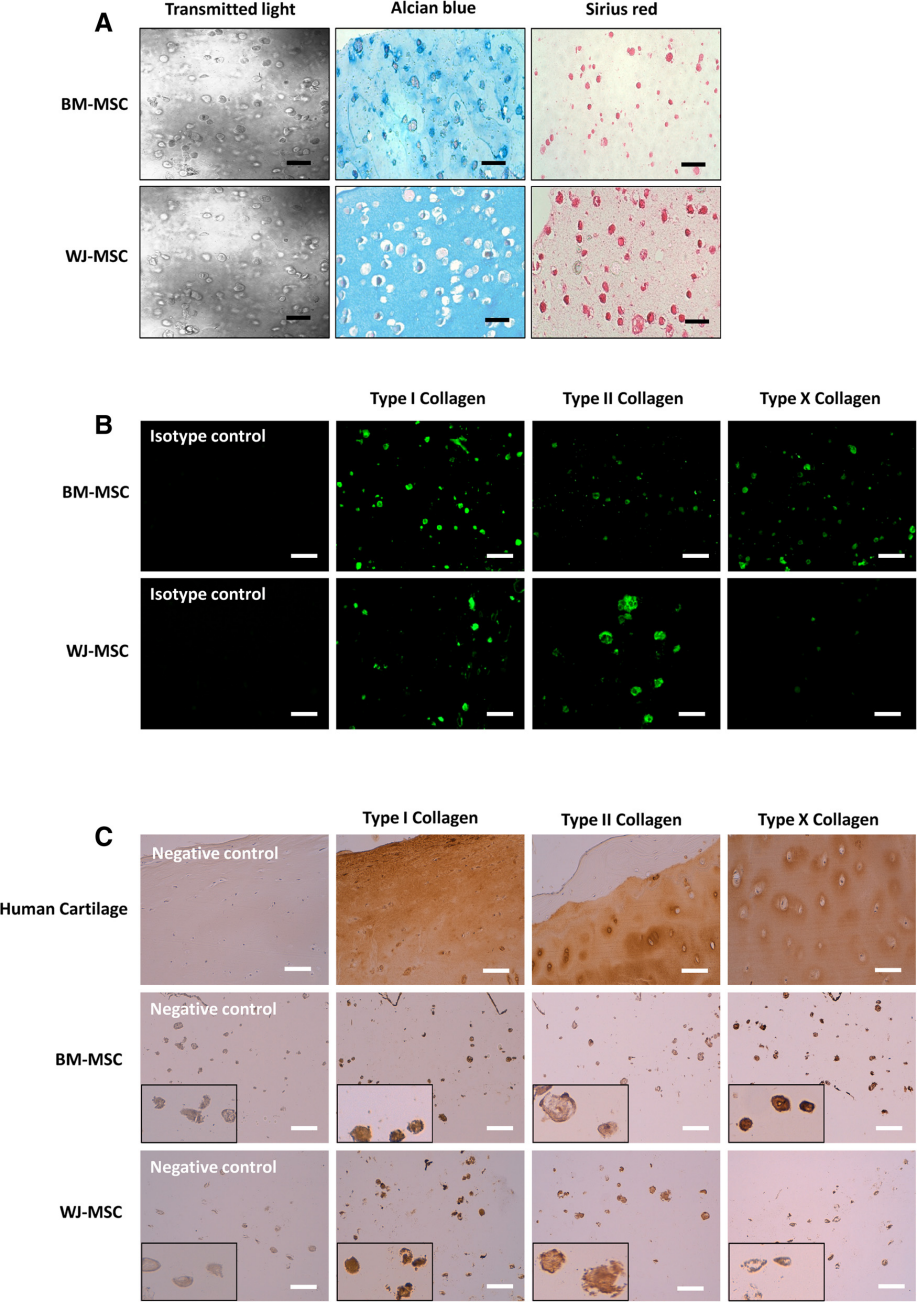
Matrix synthesis detected after 28 days of chondrogenic induction. Proteoglycans and total collagen were stained by Alcian blue and Sirius red (**a**), respectively. To explore the synthesis of various collagens in depth, immunofluorescence (**b**) and immunohistochemistry staining (**c**) were performed and detected using fluorescence microscopy and light microscopy, respectively; scale bar = 100 μm. *BM-MSC* bone marrow-derived mesenchymal stromal/stem cells, *WJ-MSC* Wharton’s jelly-derived mesenchymal stromal/stem cells

To study chondrogenic differentiation, matrix synthesis was detected after 28 days of induction. Proteoglycans and total collagen were stained by Alcian blue and Sirius red (Fig. 5a), respectively. According to histological labeling, matrix synthesis remained pericellular and total collagen synthesis seemed to be greater in WJ-MSC compared to BM-MSC. To explore the synthesis of various collagens in depth, immunofluorescence and immunohistochemistry staining were performed after 28 days of chondrogenic induction (Fig. 5b, c). Regardless of MSC source, collagen synthesis was pericellular and, as shown in Fig. 5c, remained low compared to positive control (human cartilage). Both cell types expressed type I collagen. In contrast, WJ-MSC seemed to synthesize more type II collagen, whereas BM-MSC seemed to produce more type X collagen. These results obtained for matrix synthesis analysis were consistent with those obtained for transcript analysis.

## Discussion

Today, thanks to their multiple properties and ease of access, WJ-MSC seem to be an interesting source of MSC for cartilage tissue engineering. The aim of this work was to evaluate the chondrogenic potential of WJ-MSC embedded in Alg/HA hydrogel, constructed by an original spraying method, without adding growth factors and to compare the results with those obtained with standard BM-MSC. The culture model used in this study has already been validated in previous studies [6, 20]. We showed that the spraying process contributes to form a highly functional and structured biomaterial for cartilage tissue engineering. In this present study, we investigated the potential for chondrogenic differentiation of WJ-MSC maintained in scaffolds built by spraying process. We then compared chondrogenic potential to that obtained from BM-MSC. We focused on cell viability, cell phenotype and evaluation of matrix synthesis during chondrogenic differentiation.

Alginate hydrogel is considered a natural, polysaccharide-based and biocompatible scaffold which creates a porous microstructure and is therefore a viable environment for cell culture [22]. Adult stem cells encapsulated in alginate hydrogel exhibited viability greater than 95 % up to 28 days of culture [23]. Very few studies have shown the impact of seeding WJ-MSC in alginate hydrogel on cell viability [24, 25]. Penolazzi et al. indicated that WJ-MSC, encapsulated in alginate microbeads, were highly viable (about 90 %) after 6 days of culture [24]. In the present study, we demonstrated an increase of sprayed WJ-MSC viability (>80 %) at day 14 which was maintained until the end of the culture. Few apoptotic and necrotic cells found at day 3 seemed to be due to the spraying method, and were eliminated in the last step of necrosis. This could explain the increase in sprayed cell viability. However, in further work, it would be interesting to evaluate cell number (DNA content) in hydrogel during differentiation. These results are consistent with those of a previous study which used BM-MSC under the same conditions [6]. It was demonstrated that spraying method effects are quite damaging to cell viability and metabolic activity during the first 7 days of culture [6]. To confirm our hypothesis and explain cell mortality at day 3, we performed two methods of scaffold construct: alginate beads and alginate cylinders (obtained by spraying method) seeded with the same WJ-MSC (Fig. 2c). The results indicated clearly that at the third day of culture, viability of sprayed cells was significantly lower than in cells embedded in alginate beads. At the same time, cell apoptosis was significantly higher in cylinders compared to cells seeded in beads. Thus, we have shown that the spraying method can transiently alter cell viability only during the onset of culture. This could be explained by the fact that the impact of cells on the support may induce the death of some cells. After 3 days of culture, WJ-MSC seemed to be adapted to their new 3D environment without any detectable damage. Thus, these data are consistent with previous reports [6, 20] and confirm that, even after the spraying process, Alg/HA hydrogel remains an in vitro biocompatible environment for 3D cell culture.

Cell characterization was performed by the study of surface marker expression and analysis of Sox9 transcription factor expression which are involved in chondrogenic differentiation. During monolayer culture, WJ-MSC expressed MSC surface markers (such as CD44, CD73, CD90, CD105 and CD166) as reported in previous studies [12, 26]. A very large number of WJ-MSC (almost 100 %) were positive for CD73, CD90, CD105 and it was demonstrated that expression of these markers by adult MSC was clearly in favor of a chondrogenic potential [27–29]. In monolayer and in comparison with BM-MSC, the proportion of CD44^+^, CD73^+^ and CD105^+^ WJ-MSC were significantly higher. Upregulation of the HA receptor (CD44) in WJ-MSC has been previously described and probably results in the histological structure of the umbilical cord matrix [30]. Indeed, according to histochemical analysis, Wharton’s jelly contains significant amounts of HA [31]. Moreover, it is well known that scaffold composed of HA may create an environment that can preserve the normal phenotype of chondrocytes and may guide cell differentiation to chondrogenesis [19]. A recent study showed that human MSC interactions with HA hydrogels via CD44 and CD168 promote chondrogenesis and that specific cell–material interactions play a role in this process. Beyond matrix interactions, cadherin molecules, a family of transmembrane glycoproteins, play a critical role in tissue development during embryogenesis, and N-cadherin is a key factor in mediating cell–cell interactions during mesenchymal condensation and chondrogenesis [32]. In our study, due to the presence of HA in alginate hydrogel, regardless of the MSC source and during 3D culture, CD44 expression remained high and stable as mentioned in the literature [18]. On the other hand, Lee et al. showed that in alginate hydrogel, without hyaluronic acid, a marked decrease of CD44 expression by BM-MSC was observed after 2 weeks of culture [33]. CD73, an ecto-5’-nucleotidase which plays a crucial role in extracellular adenosine generation, is known to be a regulatory factor in chondrogenic differentiation [34]. CD105 (endoglin), a membrane glycoprotein which is part of the TGFβ receptor complex [35], is considered a marker of stem cell chondrogenic potential [36]. Moreover, it has been demonstrated that the expression of these two markers decreases during chondrogenesis [33]. Thus, according to this phenotype profile (particularly CD73 and CD105 expression), before seeding in a 3D scaffold, WJ-MSC should have better chondrogenic potential compared to BM-MSC. After cell embedding in Alg/HA hydrogel and throughout culture, WJ-MSC undergo significant phenotypic changes. From 14 and up to 28 days, the percentages of positive WJ-MSC for mesenchymal markers (except CD44) decreased significantly compared to monolayer marker expression. These results are consistent with those of a previous study showing similar data with BM-MSC seeded in alginate hydrogel and cultivated with or without added growth factor for 2 weeks [33]. In 3D culture, mesenchymal marker expression levels decreased during the chondrogenic differentiation process [33]. Nagase et al. showed that CD90 was an important indicator of chondrogenic differentiation potential of synovial MSC [37]. Decreased CD90 expression was correlated with reduced chondrogenic potential [37]. According to our results, decreased expression of mesenchymal markers could reflect a loss of undifferentiated character of WJ-MSC and cell commitment to a differentiation pathway. In our study, after 14 days of 3D culture, WJ-MSC and BM-MSC showed different phenotype profiles. WJ-MSC positivity for mesenchymal markers seemed to decrease compared to BM-MSC, notably for CD90 and CD166 which were significantly reduced. To confirm cell commitment to a differentiation pathway, and particularly to chondrogenesis, we analyzed the expression of the critical chondrogenic marker, Sox9 transcription factor. Chondrogenic gene Sox9 was observed during in vitro induction of chondrogenesis and promoted transcription of cartilage protein such as type II collagen and aggrecan [2, 38]. Our data showed that the relative expression of Sox9 was highly increased between the 14th and 28th days of scaffold culture and demonstrated WJ-MSC commitment to chondrogenesis. A recent study using WJ-MSC seeded in type I collagen hydrogel, but with a chondrogenic induction by growth factor (TGF-β1), described an early upregulation of Sox9 from the day 7 of culture [13]. Also, it is known that the TGFβ receptor, via the Smad 2/3 pathway, activated the Sox9 transcription factor [38].

Evaluation of matrix synthesis was evaluated at both transcript and protein levels. Transcript expression was only evaluated after 3 days of differentiation. In future studies it would be interesting to perform day 0 transcript expression to have a real control. Cartilage matrix is composed of a network of specific molecules which give the tissue its functional properties and notably its resistance to mechanical stress [39]. After 28 days of scaffold culture, our results demonstrated a strong upregulation of cartilage-specific transcript expression. This result is consistent with those of previous studies using WJ-MSC embedded in a 3D environment and stimulated by growth factors [13, 15]. Type II collagen is the main collagen found in physiological hyaline cartilage and provides its tensile strength [39]. According to our results, after 4 weeks of chondrogenic differentiation WJ-MSC exhibited greater type II collagen synthesis than BM-MSC, at both transcript and protein levels. In a relevant study, Wang et al. demonstrated that WJ-MSC seeded on polyglycolic acid scaffold, using growth factor, synthesized nearly three times as much collagen production as BM-MSC. However, the differentiation profile of WJ-MSC, compared to BM-MSC, consisted of a larger amount of type I collagen and a smaller amount of type II collagen [40]. Based on our data, Alg/HA hydrogel scaffold seems to be a suitable environment promoting type II collagen synthesis by WJ-MSC. The fact that type IIa procollagen, considered a chondroprogenitor cell marker, was still present at 28 days seems to indicate incomplete or slow chondrogenesis in WJ-MSC [41]. No significant difference in proteoglycans synthesis was observed between WJ-MSC and BM-MSC. Furthermore, our work highlights a relevant result showing that WJ-MSC express type X collagen at lower levels than BM-MSC, at both transcript and protein levels and especially at 28 days. Type X collagen is considered a relevant hypertrophic chondrocyte marker [38] and serves as a framework for subsequent matrix calcification [42]. Hypertrophic extracellular matrix is functionally different to physiological hyaline cartilage and tends to become similar to bone matrix. Hypertrophic chondrocytes are associated with the final stage of MSC chondrogenesis [2]. Moreover, our results showed that the osteogenic transcription factor Runx2, also considered as a hypertrophic chondrocyte marker, was not expressed by WJ-MSC throughout culture compared to BM-MSC. A major challenge for cartilage tissue engineering is to control hypertrophic differentiation and maintain a stable chondrocyte phenotype [38]. In this context, according to our results, after 28 days of chondrogenic differentiation, WJ-MSC seem to be less committed towards hypertrophy than BM-MSC. Finally, it is known that MSC are able to differentiate into other mesodermic lineages such as adipogenic differentiation. Therefore, we reported that, regardless of MSC source, no expression of adipogenic transcription factor PPARγ was detected during hydrogel culture.

## Conclusions

Although numerous studies have shown the successful chondrogenic induction of WJ-MSC in 3D scaffolds, our work demonstrated chondrogenic differentiation of WJ-MSC without adding growth factors. Once seeded in Alg/HA hydrogel scaffold, WJ-MSC and BM-MSC have different profiles of chondrogenic differentiation at both transcript and protein levels. After 4 weeks, WJ-MSC, embedded in a 3D environment, were able to adapt to their environment and express specific cartilage-related genes and matrix proteins. Due to their chondrogenic differentiation potential, their low immunogenicity and the fact that they are easy to obtain, today WJ-MSC represent a real alternative source of stem cells for cartilage tissue engineering. In order to improve mimicking of the physiological environment and enhance chondrogenic differentiation, it would be interesting to apply mechanical stress [43], hypoxic culture [44] and turn to stratified cartilage tissue engineering. Our original spraying method could be used to design new stratified engineered tissues through progressive cells and hydrogel layers spraying. In all events, cartilage repair based on WJ-MSC embedded in Alg/HA hydrogel remains to be examined further by preclinical and clinical in vivo assays.

## Abbreviations

3D: Three-dimensional
Alg/HA: Alginate/hyaluronic acid
BM-MSC: Bone marrow-derived mesenchymal stromal/stem cells
BMP: Bone morphogenic protein
COMP: Cartilage oligo matrix protein
FITC: Fluorescein isothiocyanate
HA: Hyaluronic Acid
HBSS: Hanks’ balanced salt solution
MEM: Minimal Eagle medium
MSC: Mesenchymal stromal/stem cells
P: Passage
PCR: Polymerase chain reaction
PE: Phycoerythrin
PI: Propidium iodide
TGF: Transforming growth factor
WJ-MSC: Wharton’s jelly-derived mesenchymal stromal/stem cells.
